# The prevalence of masticatory muscle pain in healthy individuals with class I and class II malocclusion: a cross-sectional study

**DOI:** 10.3389/fneur.2025.1564647

**Published:** 2025-05-09

**Authors:** Małgorzata Gałczyńska-Rusin, Zofia Maciejewska-Szaniec, Małgorzata Pobudek-Radzikowska, Krzysztof Gawriołek, Agata Czajka-Jakubowska

**Affiliations:** Department of Orthodontics and Temporomandibular Disorders, Poznan University of Medical Sciences, Poznan, Poland

**Keywords:** muscle pain, malocclusion, orthodontic patients, TMD, class II

## Abstract

**Introduction:**

The etiopathogenesis of TMD is complex and involves multiple factors. The role of occlusal abnormalities in the painful form of TMD remains controversial. This study aimed to determine the prevalence of myalgia in patients with class I and class II malocclusion.

**Materials and methods:**

A total of 256 generally healthy patients, aged 25–30, with class I and class II malocclusion, were examined. Medical histories and physical examinations were conducted using the DC/TMD Form. Based on the clinical findings, the patients were divided into three groups: Group I consisted of patients with class I malocclusion; Group II included patients with class II malocclusion and proclined incisors; and Group III comprised patients with class II malocclusion and retruded incisors. Within each group, cases with muscle pain and those without were identified based on the data from the DC/TMD Form.

**Results:**

All studied groups (Groups I, II, and III) showed a high incidence of myalgia. However, statistical analysis did not show a significant difference in the overall occurrence of muscle pain between the groups, nor were there significant differences in pain incidence when examining individual muscles among the groups.

**Conclusion:**

No association was found between malocclusion and the occurrence of muscle pain. However, the more frequent presence of symptoms related to functional disorders, such as myalgia, highlights the need for screening and treatment even in generally healthy patients.

## 1 Introduction

Recent research highlights the growing complexity of temporomandibular disorders (TMD), demonstrating that their etiology extends beyond biomechanical factors to involve a multifaceted interplay of biological, psychological, and social influences (1). While TMD symptoms require treatment in approximately 15–20% of adults, only 2–4% of the population actively seek specialized care. Long-term studies indicate that TMD symptoms tend to worsen with age. Although the highest prevalence occurs between 30 and 40 (2, 3), initial symptoms requiring intervention can appear as early as childhood (4).

Significant attention has been devoted to understanding the potential cause-and-effect relationships between occlusal abnormalities and TMD-related disorders (5–8). Occlusion, traditionally defined as the static or dynamic relationship between maxillary and mandibular teeth, is now recognized as part of a more intricate, neurologically regulated system. This system integrates sensory input from oral receptors, particularly those within the periodontium and soft tissues, which is processed by the central nervous system to coordinate precise jaw movements (9).

Among TMD subtypes, myogenic TMD is the most prevalent and represents a debilitating condition (10). Patients with malocclusions commonly report muscle pain, and some studies suggest a higher incidence of muscle-related complaints among individuals with Class II malocclusion (Angle classification) (11). However, conflicting evidence exists, as other studies do not support this association, emphasizing the need for further research on this topic (12).

Given the ongoing debate regarding the relationship between malocclusion and muscle-related TMD symptoms, this study aims to assess the prevalence of masticatory muscle complaints identified through palpation among patients with Angle Class I and Class II malocclusion. This approach seeks to clarify potential correlations and contribute to a more comprehensive understanding of the interplay between occlusion and myogenic TMD.

## 2 Materials and methods

The study involved 256 participants selected from a total pool of 324 patients seeking dental treatment at two private clinics. The sample consisted of individuals of both sexes, including 179 women and 77 men, aged between 25 and 30 years. Participants were generally in good health and had no prior diagnosis or treatment for any form of temporomandibular disorder (TMD).

The exclusion criteria for the study included a history of orthodontic treatment, missing teeth in the support zones, pregnancy or lactation, neoplastic diseases, chronic systemic conditions, mental health disorders, and prior injuries to the craniofacial region.

Following a thorough review of data collected using the DC/TMD Axis I form, 256 patients were selected and deemed eligible for inclusion in the study.

All examinations were conducted by the same doctor under same conditions. Patients were positioned in a dental chair designed to promote relaxation during the examination and allow free jaw movement. They rested their heads on the headrest and their elbows on the armrests. The components of the temporomandibular system were assessed without any additional load, with the muscles in a passive state.

Muscle assessment according to the DC/TMD protocol included: temporal muscles, masseter muscles, posterior mandibular region, submandibular region, lateral pterygoid area and temporalis tendon (13, 14).

The study population was divided into three groups based on similar abnormalities in the assessed parameters:

Group I

This group consisted of 105 patients (65 women, 40 men) with the following characteristics:

- Crowding in the anterior segment.
- Canine positioning classified as Class I or incomplete Class I due to canine distortion.
- Molar positioning classified as Angle’s Class I.
- Overjet ranging from 1 to 4 mm.
- Correct transverse relationship of the buccal cusps of the molars.

Group II

This group included 99 patients (76 women, 23 men) with the following features:

- Proclined upper incisors.
- Incorrect positioning of the canines (Canine Class II) and molars (Angle’s Class II).
- Overjet ranging from 1 to 5 mm and in some cases from 4 to 7 mm.
- Correct transverse relationship of the buccal cusps of the molars.

Group III

This group comprised 52 patients (38 women, 14 men) with the following abnormalities:

- Retroclined upper incisors.
- Incorrect positioning of the canines (Canine Class II) and molars (Angle’s Class II).
- Overjet ranging from 2 to 8 mm and, in some cases, 0 mm.
- Correct transverse relationship of the buccal cusps of the molars.

Subgroups

In each study group, two subgroups were identified:

1. Painful subgroup (Id, IId, IIId): Patients presenting clinical symptoms of myalgia.
2. Control subgroup (Ic, IIc, IIIc): Patients without any clinical symptoms of muscle pain.

### 2.1 Statistics

Normality of the distribution of quantitative variables in the analyzed subgroups was checked using the Shapiro–Wilk test, visual evaluation of histograms and the levels of skewness and kurtosis of the data. Equality of variances in the subgroups was checked using the Bartlett test. When the assumptions of parametric tests were met (equality of variances, distribution close to normal), the comparison of subgroups for quantitative variables was performed using the Student’s t-test. In cases with a high level of skewness or kurtosis (above the level of 2 in absolute value), the analysis was performed using the Student’s t-test and repeated with a nonparametric test (Mann Whitney U test), which confirmed the results obtained with the Student’s t-test in each situation. All statistical tests performed were two-sided and the significance level of 0.05 was assumed for them. The analysis was performed in the R statistical package, version 3.5.1.

The study received the approval of the Bioethics Committee of the Medical University of Poznań nr 307/22.

## 3 Results

No statistically significant differences were found in terms of gender and age distribution in the individual research groups.

In each of the studied groups, the frequency of pain during palpation of the masticatory muscles was similar. No statistically significant differences were found between the studied groups. However, it should be emphasized that regardless of occlusal contacts, pain was found in approximately 50% of the examined patients (Table 1). Considering all groups, masticatory muscle pain was reported by 131 individuals, including 110 women (84%) and 21 men (16%).

**Table 1:** Analysis of the frequency of pain symptoms between groups (chi-square test).

Patient group	Group I		Group II		Group III		*p*
	n	%	n	%	n	%	
Patients with pain in the muscles under palpation	53	50.5	52	52.5	26	46.2	0.349
Patients without muscle pain	52	49.5	47	47.5	28	53.8	0.881

Next, the frequency of symptoms of dysfunction of individual muscles was analyzed between the studied groups of patients. In group I, the lateral pterygoid area was the most frequently affected muscle (32.4%), followed by the temporalis tendon (26.7%) and the masseter (25.7%). In group II, pain in the masseter (25.3%) and temporalis tendon (24.2%) were similarly common, with the lateral pterygoid area also highly affected (23.2%). In group III, the masseter was the most frequently affected muscle (30.8%), followed by the temporalis muscle (19.2%) and posterior mandibular region (17.3%).

There were no statistically significant differences (*p* > 0.05) in the frequency of clinical dysfunction symptoms in any of the analyzed muscle types between the studied groups. The detailed results are presented in Table 2.

**Table 2:** Analysis of the frequency of pain symptoms in individual muscles between groups.

Muscle	Group I		Group II		Group III		*p*
	n	%	n	%	n	%	
Masseter	27	25.7	25	25.3	16	30.8	0.742
Temporalis	11	10.5	18	18.2	10	19.2	0.207
Posterior mandibular region	12	11.4	11	11.1	9	17.3	0.501
Submandibular region	11	10.5	9	9.1	6	11.5	0.885
Lateral pterygoid area	34	32.4	23	23.2	9	17.3	0.096
Temporalis tendon	28	26.7	24	24.2	9	17.3	0.429

## 4 Discussion

The relationship between malocclusion and functional disorders of the masticatory system (TMD) has long attracted researchers’ interest. However, the results of the studies thus far remain inconclusive (12). Some authors emphasize that malocclusion can impact the biomechanics of the masticatory system, leading to muscle and temporomandibular joint (TMJ) overload (12, 15).

Patients with Angle Class II malocclusion exhibit distinct electromyographic (EMG) patterns, including increased activity in the masseter and temporalis muscles during clenching, suggesting altered muscle function (16). Additionally, individuals with Angle Class II malocclusion frequently report a higher prevalence of muscle-related complaints, as highlighted by Farronto (17).

Conversely, many authors emphasize the multifactorial etiology of TMD, in which malocclusion is only one of several potential risk factors (1, 18, 19). Some studies have reported cases where orthodontic treatment improved TMD symptoms, suggesting occlusion may influence these disorders (20). However, other studies indicate that orthodontic therapy does not continually improve symptoms, emphasizing the complexity of the relationship between malocclusion and TMD (21). While biomechanical analyses suggest that occlusal asymmetries can contribute to muscle dysfunction, the exact mechanism remains unclear and requires further investigation (22).

Modern research increasingly incorporates systemic and psychological factors that may modulate the impact of malocclusion on the development of TMD. Simultaneously, there is a growing emphasis on the need for standardization in assessing malocclusion and diagnosing TMD, which would facilitate a more precise evaluation of their interrelationship (12). However, the wide variability in diagnostic techniques and malocclusion classifications poses a significant challenge, making cross-study comparisons difficult.

Several studies have explored the association between Class II malocclusion and TMD. For example, Szentpétery et al. observed a significantly higher incidence of TMD in patients with Class II/2 malocclusion and deep bite (23). Similarly, Bertoli et al. reported that individuals with Angle Class II malocclusion experienced myofascial pain more frequently than those with Angle Class I malocclusion (5). Uetanabaro found that 32% of adult patients with skeletal Class II malocclusion requiring orthognathic surgery presented with myofascial pain (24). Furthermore, Angelo’s research indicated that compared to Class I, Class II malocclusion was associated with greater TMD severity, increased myalgia, more frequent occurrences of disk displacement without reduction, and reduced maximum mouth opening (11).

In our study, all patients in Group III (Class II/2 malocclusion) were characterized by a deepened vertical overlap of the incisors ranging from 4.00 to 8.00 mm. However, statistical analysis did not reveal significant differences in dysfunction prevalence compared to the control group. In contrast, Tsolka et al. found that Class II/2 malocclusions were more common in individuals without TMD symptoms (46%) than in those with symptoms, where Class I predominated (38%) (25). Based on our study findings, no statistically significant differences were observed in the frequency of muscle pain between Class I and Class II malocclusions.

Furthermore, no significant relationship was found between occlusal parameters and the presence of muscle pain in our study. This suggests that dental abnormalities characteristic of Class II malocclusion are unlikely to contribute to pain development during muscle palpation. Consequently, our findings do not support a direct relationship between Angle Class II malocclusion and masticatory muscle pain, a conclusion that aligns with Emes et al. (26). However, given the high incidence of palpation-induced muscle pain, incorporating routine palpation assessments into clinical examinations could be valuable for diagnosing pain-related disorders. Such an approach should also be considered a standard practice in everyday dental care (27). Further research is essential to confirm the effectiveness of this diagnostic strategy, optimize its clinical application, and ultimately improve diagnostic accuracy and patient outcomes.

In Groups I and II, the most commonly reported symptoms were pain in the masseter muscles, lateral pterygoids, and temporalis tendon. According to Meada-Iino, palpation-induced pain in the masseter muscles is associated with increased clenching activity, characterized by a higher frequency and prolonged duration of tonic bursts (28). The masseter muscle’s location and structure make assessing it relatively easy, ensuring diagnostic accuracy. However, the diagnostic value of lateral pterygoid muscle palpation remains controversial (29, 30). Studies indicate that palpation in the lateral pterygoid region lacks sufficient specificity, necessitating caution when interpreting positive findings (30). Nonetheless, we included lateral pterygoid palpation in our study, which is an integral part of the DC/TMD examination protocol.

Additionally, assessment of the temporalis tendon is important, as tendinosis in this region can be a source of orofacial pain (31). According to Bressler, temporal tendinosis is often overlooked and underdiagnosed, reinforcing the need for its evaluation in clinical practice (31). In Group III, the masseter muscle was the most reported pain site, with other muscle regions exhibiting a similar symptom frequency.

This study challenges the long-standing belief that malocclusions are directly linked to painful TMD, emphasizing that occlusion alone is not a sufficient predictor of masticatory muscle pain. By highlighting the risk of overtreatment and unnecessary costs, our findings underscore the need for a more evidence-based approach to diagnosing and managing TMD in orthodontic and dental practice.

## 5 Limitations

One limitation of this study is that it did not include an assessment of psychological factors such as stress, parafunctional habits, and psychological conditions, which are known to influence TMD symptoms potentially. Future research should incorporate these aspects to provide a more comprehensive analysis. Additionally, Group III (Class II/2 malocclusion with retruded incisors) had a smaller sample size than the other groups, which may have influenced the study’s statistical power. However, this reflects the natural distribution of malocclusion types in the studied population.

## 6 Conclusion

In conclusion, although Angle Class II malocclusion may influence masticatory muscle function and activity, it cannot be considered a direct cause of muscle pain. The development of TMD-related pain is multifactorial, and occlusion alone is not a sufficient predictor of its presence. This underscores the complexity of TMD diagnosis and treatment, emphasizing the need for a comprehensive, multidisciplinary approach to patient evaluation.

## Data Availability

The raw data supporting the conclusions of this article will be made available by the authors, without undue reservation.
